# Crystal structure of 2-(1-methyl­eth­yl)-1,3-thia­zolo[4,5-*b*]pyridine

**DOI:** 10.1107/S2056989015006039

**Published:** 2015-04-02

**Authors:** Gamal A. El-Hiti, Keith Smith, Amany S. Hegazy, Saud A. Alanazi, Benson M. Kariuki

**Affiliations:** a Cornea Research Chair, Department of Optometry, College of Applied Medical, Sciences, King Saud University, PO Box 10219, Riyadh 11433, Saudi Arabia; b School of Chemistry, Cardiff University, Main Building, Park Place, Cardiff CF10 3AT, Wales

**Keywords:** crystal structure, thia­zolo­pyridine, hydrogen bonding

## Abstract

In the title mol­ecule, C_9_H_10_N_2_S, one of the methyl groups is almost co-planar with the thia­zolo­pyridine rings with a deviation of 0.311 (3) Å from the least-squares plane of the thia­zolo­pyridine group. In the crystal, weak C—H⋯N hydrogen-bonding inter­actions lead to the formation of chains along [011].

## Related literature   

For related compounds, see: Smith *et al.* (1994 ▸, 1995 ▸); El-Hiti (2003 ▸); Johnson *et al.* (2006 ▸); Thomae *et al.* (2008 ▸); Rao *et al.* (2009 ▸); Lee *et al.* (2010 ▸); Luo *et al.* (2015 ▸). For the X-ray crystal structures of related compounds, see: Yu *et al.* (2007 ▸); El-Hiti *et al.* (2014 ▸).
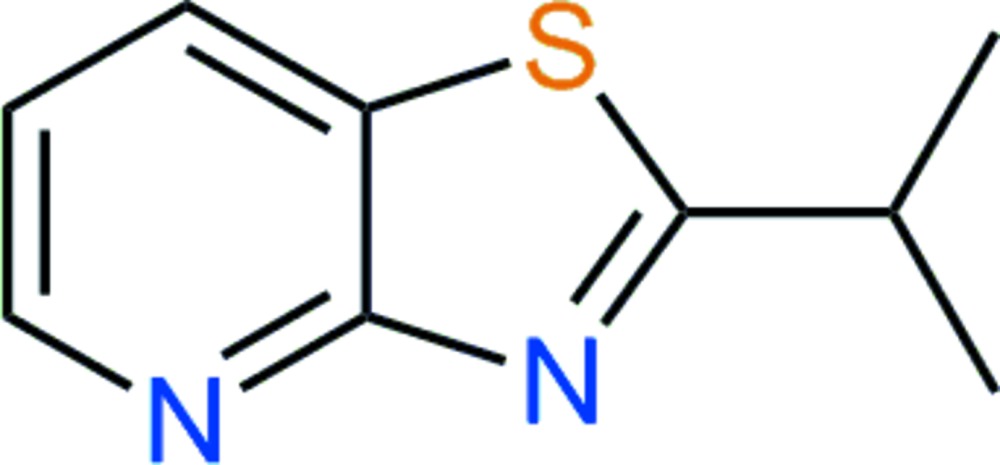


## Experimental   

### Crystal data  

C_9_H_10_N_2_S
*M* *_r_* = 178.25
Orthorhombic, 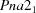

*a* = 9.6376 (2) Å
*b* = 10.1602 (2) Å
*c* = 8.9254 (2) Å
*V* = 873.98 (3) Å^3^
*Z* = 4
Cu *K*α radiation
μ = 2.81 mm^−1^
*T* = 150 K
0.23 × 0.20 × 0.14 mm

### Data collection  

Agilent SuperNova, Dual, Cu at zero, Atlas diffractometer
Absorption correction: multi-scan (*CrysAlis PRO*; Agilent, 2014 ▸) *T*_min_ = 0.897, *T*_max_ = 0.940
2848 measured reflections
1366 independent reflections
1351 reflections with *I* > 2σ(*I*)
*R*_int_ = 0.012

### Refinement  

*R*[*F* ^2^ > 2σ(*F* ^2^)] = 0.021
*wR*(*F* ^2^) = 0.057
*S* = 1.08
1366 reflections
111 parameters
1 restraint
H-atom parameters constrained
Δρ_max_ = 0.19 e Å^−3^
Δρ_min_ = −0.20 e Å^−3^

### 

Data collection: *CrysAlis PRO* (Agilent, 2014 ▸); cell refinement: *CrysAlis PRO*; data reduction: *CrysAlis PRO*; program(s) used to solve structure: *SHELXS2013* (Sheldrick, 2008 ▸); program(s) used to refine structure: *SHELXL2013* (Sheldrick, 2015 ▸); molecular graphics: *ORTEP-3 for Windows* (Farrugia, 2012 ▸); software used to prepare material for publication: *WinGX* (Farrugia, 2012 ▸) and *CHEMDRAW* Ultra (Cambridge Soft, 2001 ▸).

## Supplementary Material

Crystal structure: contains datablock(s) I, New_Global_Publ_Block. DOI: 10.1107/S2056989015006039/zs2329sup1.cif

Structure factors: contains datablock(s) I. DOI: 10.1107/S2056989015006039/zs2329Isup2.hkl

Supporting information file. DOI: 10.1107/S2056989015006039/zs2329Isup3.cml

**Fig. 1.**
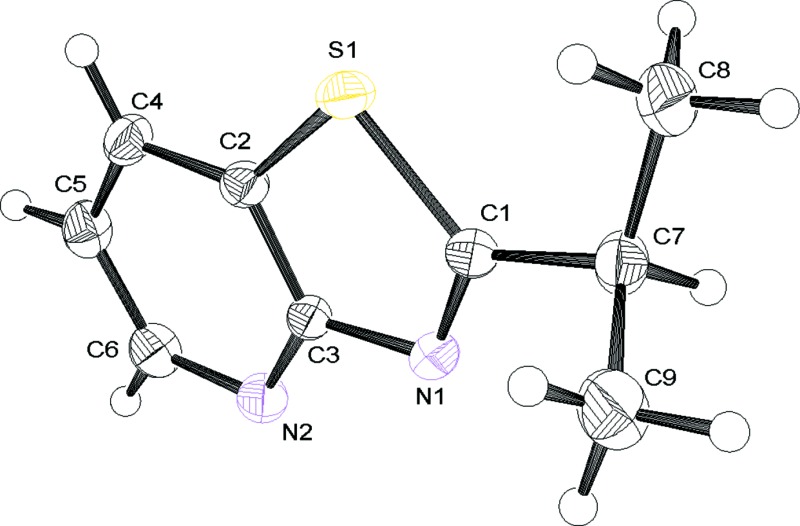
A molecule of C9H10N2S with atom labels and 50% probability displacement ellipsoids for non-hydrogen atoms.

**Fig. 2.**
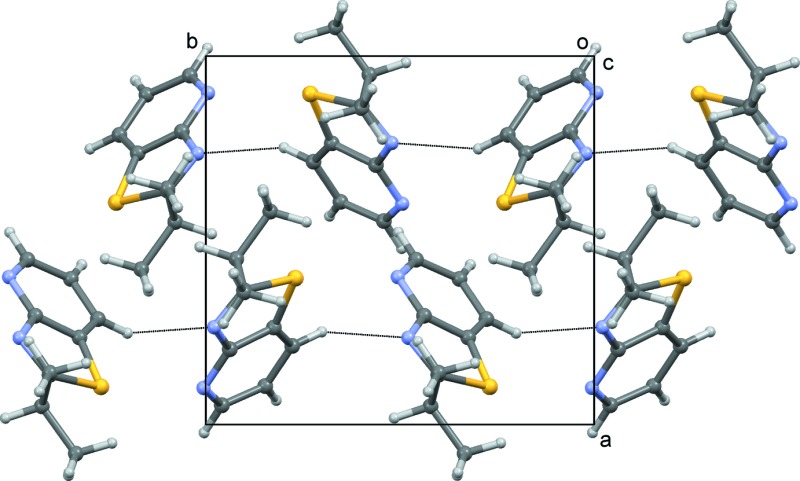
Crystal structure packing viewed down the c axis with C—H···N interactions shown as dotted lines.

9 10 2 . DOI: 10.1107/S2056989015006039/zs2329fig1.tif
A mol­ecule of C_9_H_10_N_2_S with atom labels and 50% probability displacement ellipsoids for non-hydrogen atoms.

c . DOI: 10.1107/S2056989015006039/zs2329fig2.tif
Crystal structure packing viewed down the *c* axis with C—H⋯N inter­actions shown as dotted lines.

CCDC reference: 1056012

Additional supporting information:  crystallographic information; 3D view; checkCIF report

## Figures and Tables

**Table 1:** Hydrogen-bond geometry (, )

*D*H*A*	*D*H	H*A*	*D* *A*	*D*H*A*
C4H4N1^i^	0.95	2.51	3.391(2)	153

Symmetry code: (i) 
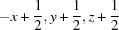
.

## supplementary crystallographic information

### S1. Chemical context

Thia­zolo­pyridines have been efficiently synthesized and in high yield using different synthetic procedures (Smith *et al.*, 1994, 1995; El-Hiti, 2003; Johnson *et al.*, 2006; Thomae *et al.*, 2008; Rao *et al.*, 2009; Lee *et al.*, 2010; Luo *et al.*, 2015). During our continuing research towards the development of novel synthetic routes for the production of heterocyclic compounds, we have synthesised the title compound 2-(methyl­ethyl)-1,3-thia­zolo[4,5-*b*]pyridine in high yield (Smith *et al*., 1995). The X-ray structures for related compounds have been reported (Yu *et al.*, 2007; El-Hiti *et al.*, 2014).

### S2. Structural commentary

The asymmetric unit of the title compound consists of a single molecule of C_9_H_10_N_2_S (Fig. 1). In the molecule, one of the methyl groups is almost co-planar with the thia­zolo­pyridine ring. The deviations from the least-squares plane of the thia­zolo­pyridine group are 0.311 (3)Å and 1.269 (3)Å for C8 and C9 respectively, corresponding to torsion angles N1—C1—C7—C8 and N1—C1—C7—C9 of 169.47 (19) and -65.9 (3)°, respectively.

Weak C—H···N hydrogen-bonding inter­actions occur in the structure to form chains along [011] (Fig. 2, Table 1). No π–π inter­actions are observed in the crystal structure.

### S3. Synthesis and crystallization

2-(1-Methyl­ethyl)-1,3-thia­zolo[4,5-*b*]pyridine was obtained in 98% yield from acid hydrolysis (HCl, 5 M) of 3-(diiso­propyl­amino­thio­carbonyl­thio)-2-(1-methyl­ethyl­carbonyl­amino)­pyridine under reflux for 5 h (Smith *et al.*, 1995). Crystallization of the crude product from di­ethyl ether gave colourless crystals of the title compound. The spectroscopic and analytical data for the title compound were consistent with those reported previously (Smith *et al.*, 1995).

### S4. Refinement details

H atoms were positioned geometrically and refined using a riding model with *U*_iso_(H) constrained to be 1.2 times *U*_eq_ for the atom it is bonded to except for methyl groups where it was 1.5 times with free rotation about the C—C bond. Although not of relevance with this achiral compound, the absolute structure factor (Flack, 1983) was determined as 0.031 (11) for 434 Friedel pairs.

### Crystal data

**Table:**

C_9_H_10_N_2_S	*D*_x_ = 1.355 Mg m^−^^3^
*M**_r_* = 178.25	Cu *K*α radiation, λ = 1.54184 Å
Orthorhombic, *P**n**a*2_1_	Cell parameters from 2276 reflections
*a* = 9.6376 (2) Å	θ = 6.3–73.8°
*b* = 10.1602 (2) Å	µ = 2.81 mm^−^^1^
*c* = 8.9254 (2) Å	*T* = 150 K
*V* = 873.98 (3) Å^3^	Block, colourless
*Z* = 4	0.23 × 0.20 × 0.14 mm
*F*(000) = 376	

### Data collection

**Table:**

Agilent SuperNova, Dual, Cu at zero, Atlas diffractometer	1366 independent reflections
Radiation source: sealed X-ray tube, SuperNova (Cu) X-ray Source	1351 reflections with *I* > 2σ(*I*)
Mirror monochromator	*R*_int_ = 0.012
Detector resolution: 10.5082 pixels mm^-1^	θ_max_ = 74.0°, θ_min_ = 6.3°
ω scans	*h* = −11→10
Absorption correction: multi-scan (*CrysAlis PRO*; Agilent, 2014)	*k* = −12→8
*T*_min_ = 0.897, *T*_max_ = 0.940	*l* = −10→10
2848 measured reflections	

### Refinement

**Table:**

Refinement on *F*^2^	1 restraint
Least-squares matrix: full	Hydrogen site location: inferred from neighbouring sites
*R*[*F*^2^ > 2σ(*F*^2^)] = 0.021	H-atom parameters constrained
*wR*(*F*^2^) = 0.057	*w* = 1/[σ^2^(*F*_o_^2^) + (0.0375*P*)^2^ + 0.1081*P*] where *P* = (*F*_o_^2^ + 2*F*_c_^2^)/3
*S* = 1.08	(Δ/σ)_max_ = 0.001
1366 reflections	Δρ_max_ = 0.19 e Å^−^^3^
111 parameters	Δρ_min_ = −0.20 e Å^−^^3^

### Special details

**Table:**

Geometry. All e.s.d.'s (except the e.s.d. in the dihedral angle between two l.s. planes) are estimated using the full covariance matrix. The cell e.s.d.'s are taken into account individually in the estimation of e.s.d.'s in distances, angles and torsion angles; correlations between e.s.d.'s in cell parameters are only used when they are defined by crystal symmetry. An approximate (isotropic) treatment of cell e.s.d.'s is used for estimating e.s.d.'s involving l.s. planes.

### Fractional atomic coordinates and isotropic or equivalent isotropic displacement parameters (Å^2^)

**Table:**

	*x*	*y*	*z*	*U*_iso_*/*U*_eq_	
C1	0.13501 (18)	0.59856 (18)	0.8660 (2)	0.0227 (4)	
C2	0.23657 (17)	0.67547 (17)	1.1015 (2)	0.0208 (3)	
C3	0.29550 (18)	0.56187 (17)	1.0365 (2)	0.0203 (3)	
C4	0.2865 (2)	0.72261 (17)	1.2365 (3)	0.0257 (4)	
H4	0.2483	0.7985	1.2829	0.031*	
C5	0.39534 (19)	0.6532 (2)	1.3007 (3)	0.0279 (4)	
H5	0.4342	0.6813	1.3931	0.034*	
C6	0.44760 (19)	0.54145 (19)	1.2283 (3)	0.0273 (4)	
H6	0.5220	0.4959	1.2752	0.033*	
C7	0.05003 (18)	0.57871 (19)	0.7259 (3)	0.0271 (4)	
H7	0.0177	0.4852	0.7251	0.033*	
C8	−0.0783 (2)	0.6663 (2)	0.7204 (3)	0.0365 (5)	
H8A	−0.1375	0.6475	0.8070	0.055*	
H8B	−0.1299	0.6488	0.6279	0.055*	
H8C	−0.0499	0.7589	0.7227	0.055*	
C9	0.1411 (2)	0.5992 (3)	0.5875 (3)	0.0421 (6)	
H9A	0.1666	0.6923	0.5796	0.063*	
H9B	0.0897	0.5727	0.4977	0.063*	
H9C	0.2253	0.5457	0.5966	0.063*	
N1	0.23563 (16)	0.52156 (16)	0.9037 (2)	0.0241 (3)	
N2	0.40055 (14)	0.49434 (16)	1.0985 (2)	0.0254 (4)	
S1	0.10273 (4)	0.73079 (4)	0.98822 (7)	0.02515 (14)	

### Atomic displacement parameters (Å^2^)

**Table:**

	*U*^11^	*U*^22^	*U*^33^	*U*^12^	*U*^13^	*U*^23^
C1	0.0205 (8)	0.0253 (8)	0.0224 (10)	−0.0013 (7)	0.0032 (7)	−0.0026 (8)
C2	0.0212 (8)	0.0210 (7)	0.0203 (9)	−0.0011 (6)	0.0033 (6)	−0.0004 (7)
C3	0.0199 (7)	0.0206 (7)	0.0204 (9)	−0.0026 (6)	0.0032 (6)	−0.0024 (7)
C4	0.0292 (9)	0.0258 (9)	0.0221 (9)	−0.0028 (7)	0.0018 (8)	−0.0061 (8)
C5	0.0302 (9)	0.0353 (11)	0.0184 (9)	−0.0058 (7)	−0.0024 (7)	0.0002 (9)
C6	0.0258 (8)	0.0303 (9)	0.0259 (9)	0.0005 (7)	−0.0035 (8)	0.0042 (8)
C7	0.0266 (8)	0.0293 (9)	0.0254 (9)	−0.0036 (7)	−0.0032 (8)	−0.0030 (8)
C8	0.0293 (9)	0.0428 (12)	0.0374 (13)	0.0037 (9)	−0.0127 (9)	−0.0071 (11)
C9	0.0351 (11)	0.0705 (16)	0.0207 (11)	−0.0072 (11)	−0.0024 (8)	−0.0025 (11)
N1	0.0231 (7)	0.0264 (8)	0.0229 (8)	0.0012 (6)	0.0001 (6)	−0.0067 (7)
N2	0.0238 (8)	0.0239 (7)	0.0284 (9)	0.0024 (6)	−0.0016 (6)	−0.0002 (7)
S1	0.0242 (2)	0.0266 (2)	0.0246 (2)	0.00703 (13)	−0.0007 (2)	−0.0048 (2)

### Geometric parameters (Å, º)

**Table:**

C1—N1	1.291 (2)	C6—N2	1.334 (3)
C1—C7	1.508 (3)	C6—H6	0.9500
C1—S1	1.7585 (19)	C7—C8	1.524 (3)
C2—C4	1.383 (3)	C7—C9	1.530 (3)
C2—C3	1.411 (2)	C7—H7	1.0000
C2—S1	1.7325 (19)	C8—H8A	0.9800
C3—N2	1.342 (2)	C8—H8B	0.9800
C3—N1	1.380 (2)	C8—H8C	0.9800
C4—C5	1.388 (3)	C9—H9A	0.9800
C4—H4	0.9500	C9—H9B	0.9800
C5—C6	1.400 (3)	C9—H9C	0.9800
C5—H5	0.9500		
			
N1—C1—C7	122.91 (17)	C8—C7—C9	111.1 (2)
N1—C1—S1	115.73 (15)	C1—C7—H7	107.6
C7—C1—S1	121.36 (14)	C8—C7—H7	107.6
C4—C2—C3	120.08 (17)	C9—C7—H7	107.6
C4—C2—S1	130.92 (14)	C7—C8—H8A	109.5
C3—C2—S1	108.98 (14)	C7—C8—H8B	109.5
N2—C3—N1	121.16 (16)	H8A—C8—H8B	109.5
N2—C3—C2	123.53 (18)	C7—C8—H8C	109.5
N1—C3—C2	115.30 (16)	H8A—C8—H8C	109.5
C2—C4—C5	116.51 (17)	H8B—C8—H8C	109.5
C2—C4—H4	121.7	C7—C9—H9A	109.5
C5—C4—H4	121.7	C7—C9—H9B	109.5
C4—C5—C6	119.6 (2)	H9A—C9—H9B	109.5
C4—C5—H5	120.2	C7—C9—H9C	109.5
C6—C5—H5	120.2	H9A—C9—H9C	109.5
N2—C6—C5	124.73 (18)	H9B—C9—H9C	109.5
N2—C6—H6	117.6	C1—N1—C3	110.99 (16)
C5—C6—H6	117.6	C6—N2—C3	115.54 (16)
C1—C7—C8	112.90 (18)	C2—S1—C1	89.00 (9)
C1—C7—C9	109.85 (15)		
			
C4—C2—C3—N2	−0.3 (3)	C7—C1—N1—C3	−179.88 (16)
S1—C2—C3—N2	−179.06 (14)	S1—C1—N1—C3	0.6 (2)
C4—C2—C3—N1	178.46 (16)	N2—C3—N1—C1	178.62 (17)
S1—C2—C3—N1	−0.3 (2)	C2—C3—N1—C1	−0.1 (2)
C3—C2—C4—C5	0.4 (3)	C5—C6—N2—C3	0.0 (3)
S1—C2—C4—C5	178.89 (15)	N1—C3—N2—C6	−178.61 (17)
C2—C4—C5—C6	−0.4 (3)	C2—C3—N2—C6	0.1 (3)
C4—C5—C6—N2	0.2 (3)	C4—C2—S1—C1	−178.11 (19)
N1—C1—C7—C8	169.47 (19)	C3—C2—S1—C1	0.50 (14)
S1—C1—C7—C8	−11.0 (2)	N1—C1—S1—C2	−0.64 (15)
N1—C1—C7—C9	−65.9 (3)	C7—C1—S1—C2	179.79 (16)
S1—C1—C7—C9	113.64 (19)		

### Hydrogen-bond geometry (Å, º)

**Table:**

*D*—H···*A*	*D*—H	H···*A*	*D*···*A*	*D*—H···*A*
C4—H4···N1^i^	0.95	2.51	3.391 (2)	153

Symmetry code: (i) −*x*+1/2, *y*+1/2, *z*+1/2.
