# Supplementary figures and images for: Crystal structure of 2-(1-methyl­eth­yl)-1,3-thia­zolo[4,5-b]pyridine
Source: Acta Crystallogr E Crystallogr Commun. 2015 Apr 2;71(Pt 5):o272–3. doi: 10.1107/S2056989015006039 (PMC4420086; doi:10.1107/S2056989015006039)

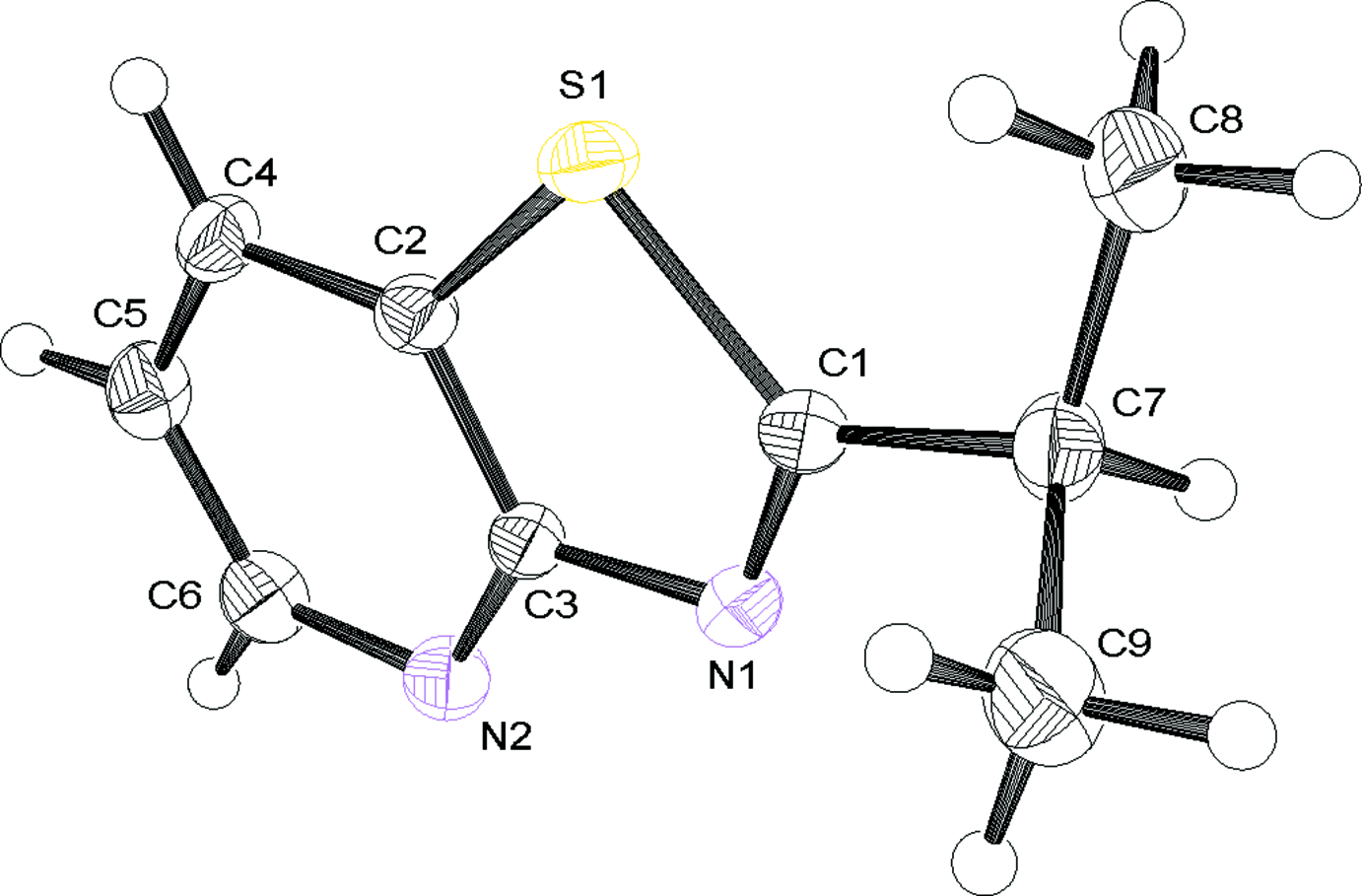

Supplement: Supplementary file 4 [file e-71-0o272-fig1.tif]

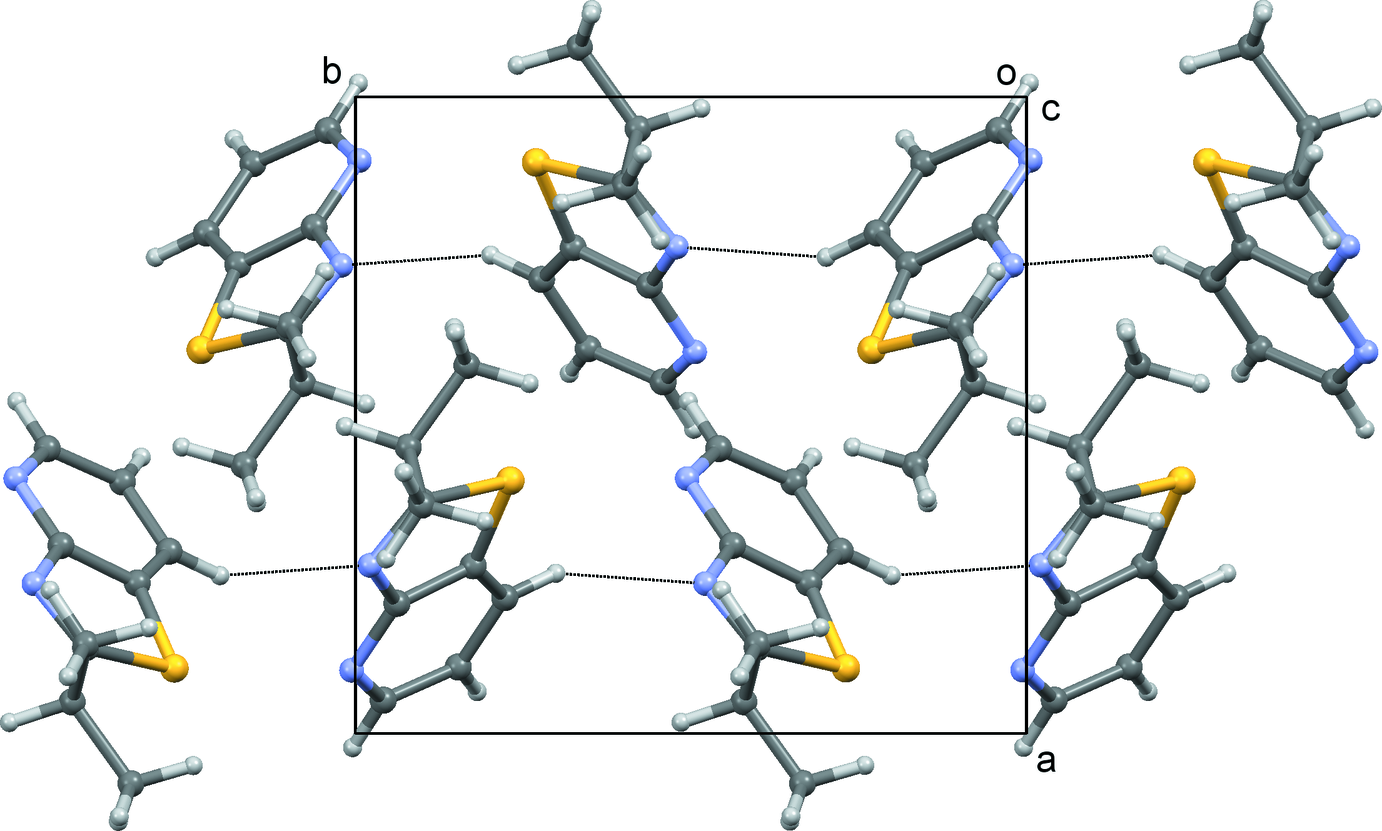

Supplement: Supplementary file 5 [file e-71-0o272-fig2.tif]
